# Preeclampsia in kidney transplanted women; Outcomes and a simple prognostic risk score system

**DOI:** 10.1371/journal.pone.0173420

**Published:** 2017-03-20

**Authors:** Guri Baardstu Majak, Anna Varberg Reisæter, Manuela Zucknick, Bjørg Lorentzen, Siri Vangen, Tore Henriksen, Trond Melbye Michelsen

**Affiliations:** 1 Department of Obstetrics, Division of Gynecology and Obstetrics, Oslo University Hospital Rikshospitalet, Oslo, Norway; 2 Norwegian National Advisory Unit on Women´s Health, Oslo University Hospital, Rikshospitalet, Oslo, Norway; 3 The Norwegian Renal Registry, Department of Transplantation Medicine, Oslo University Hospital, Rikshospitalet, Oslo, Norway; 4 Oslo Centre for Biostatistics and Epidemiology, Department of Biostatistics, Institute of Basic Medical Sciences, University of Oslo, Oslo, Norway; 5 University of Oslo, Oslo, Norway; 6 Research Unit, Sørlandet Hospital, Arendal, Norway; University Medical Center Utrecht, NETHERLANDS

## Abstract

Women pregnant following kidney transplantation are at high risk of preeclampsia. Identifying the effects of preeclampsia on pregnancy outcome and allograft function in kidney transplanted women, and predicting which women will require more targeted follow-up and possible therapeutic intervention, could improve both maternal and neonatal outcome. In this retrospective cohort study of all pregnancies following kidney transplantation in Norway between 1969 and 2013, we used medical records to identify clinical characteristics predictive of preeclampsia. 175 pregnancies were included, in which preeclampsia was diagnosed in 65. Pregnancies with preeclampsia had significantly higher postpartum serum creatinine levels, higher risks of preterm delivery, caesarean delivery, and small for gestational age infants. In the final multivariate model chronic hypertension (aOR = 5.02 [95% CI, 2.47–10.18]), previous preeclampsia (aOR = 3.26 [95% CI, 1.43–7.43]), and elevated serum creatinine (≥125 μmol/L) at the start of pregnancy (aOR = 5.79 [95% CI, 1.91–17.59]) were prognostic factors for preeclampsia. Based on this model the risk was 19% when none of these factors were present, 45–59% risk when one was present, 80–87% risk when two were present, and 96% risk when all three were present. We suggest that the risk of preeclampsia in pregnancies in kidney transplanted women can be predicted with these variables, which are easily available at the start of pregnancy.

## Introduction

Obstetric, perinatal and nephrologic care has improved since the first registered pregnancy in a kidney recipient in 1958 [1]. However, many questions still remain about how the pregnancy following kidney transplant affects the mother, child, and allograft.

Preeclampsia is one of the leading causes of maternal and perinatal morbidity and mortality worldwide. Even though the risk of preeclampsia is six times higher in kidney transplanted women compared to women without a transplant [2–4], there is little information about the direct association between preeclampsia and pregnancy outcomes in this group. For the mother and child, both preeclampsia and the need for preterm delivery have potential negative long-term health effects, including increased risks for later diseases like diabetes, adiposity and cardiovascular disease [5–7]. Preeclampsia is also associated with an increased risk for later end-stage renal disease [8] and may adversely affect the allograft and its function. In a previous study, we reported a high rate of chronic hypertension in Norwegian kidney transplanted women with preeclampsia [4]. Finding clinical characteristics that can lead to more accurate and early prediction of women at high risk of preeclampsia should be a first step in targeting prevention and follow-up. This may potentially decrease short and long-term complications.

In this study, we investigated the effects of preeclampsia on pregnancy outcome and allograft function. We also examined maternal clinical characteristics in women with pregnancies following kidney transplantation using consistent diagnostic criteria. These data were used to identify prognostic factors that can be used to predict women at risk for preeclampsia in pregnancies following kidney transplantation.

## Materials and methods

### Study design

This was a retrospective cohort study of pregnancies following kidney transplantation in Norway between 1969 and 2013. Women with pregnancies following kidney transplantation were identified by linking the Norwegian Renal Registry and the Medical Birth Registry by social security numbers. All pregnancies in Norway are reported to the Medical Birth Registry by the attending midwife or doctor, and all kidney transplantations in Norway are performed at a national center (Oslo University Hospital), and recorded in the Norwegian Renal Registry. All data used in this study were collected directly from the women’s medical records.

This study was approved by the Regional Committee for Medical Research Ethics in South-East Norway (reference number 2012/1139), is the second involving this cohort of women [4]. All women included in the study provided written informed consent permitting access to their medical records. The STROBE guidelines were followed for reporting the results of this study [9].

### Data extracted

Preeclampsia was retrospectively diagnosed in all pregnancies according to the revised recommendations of the American College of Obstetricians and Gynecologists,[10] which includes a systolic blood pressure ≥ 140 mm Hg or diastolic blood pressure ≥ 90 mm Hg after 20 weeks of gestation and proteinuria ≥ 1+ on a urine dipstick on at least two occasions. The presence of chronic hypertension (yes/no) was defined according to the revised American College of Obstetricians and Gynecologists criteria [10], which includes a systolic/diastolic blood pressure ≥ 140/90 mm Hg registered before week 20 of pregnancy and no proteinuria. In addition, all women using antihypertensive medication at the start of pregnancy were considered to have chronic hypertension. Additional subject characteristics included maternal age in years; Scandinavian ethnicity (yes/no); the presence of pre-pregnancy diabetes needing treatment with insulin or anti-diabetics (yes/no); current smoking habits (yes/no); use of immunosuppressive agents, including prednisolone (yes/no), azathioprine (yes/no), tacrolimus (yes/no), or cyclosporin (yes/no); serum creatinine (sCr) level in μmol/L before pregnancy, during first trimester, before delivery, and at a first medical check-up at least 6 weeks after delivery; and pregnancy with preeclampsia prior to kidney transplantation (yes/no). Pregnancy characteristics included the time between transplantation and pregnancy; previous pregnancy prior to transplantation (yes/no); presence of gestational diabetes (yes/no), defined as diabetes diagnosed for first time during pregnancy and treated with diet only, insulin, or anti-diabetics; twin pregnancy (yes/no); gestational age at birth in weeks; and birth weight in grams.

### Statistical methods

Pregnancies following kidney transplantation were grouped according to the presence or absence of preeclampsia. Normally distributed continuous variables were summarized as means with standard deviations and compared using a t-test. Non-normally distributed continuous variables were summarized as medians with interquartile ranges and compared using the Wilcoxon rank-sum test. Categorical variables were reported as percentages and were compared by Chi-squared test or, for small sample sizes, Fisher’s exact test. Maternal clinical features were first examined by univariate analysis using chi-square tables and odds ratios. Multivariate analysis with preeclampsia as the dependent variable was performed by logistic regression using a backward stepwise method, where removal testing is based on the probability of the likelihood-ratio statistic based on conditional parameter estimates (using default cutoff values). To account for more than one pregnancy in the same woman, robust Huber-White variance-covariance estimates were calculated in the logistic regression. Model discrimination was examined using the concordance index. The odds ratios (OR) with 95% confidence intervals (CI) were calculated for each variable. Significance was set at a p-value of less than 0.05.

Because the prognostic score did not improve significantly when sCr was used as a continuous variable, elevated sCr was defined as ≥125 μmol/L for the logistic regression to allow comparison with previous reports [11]. Linear regression with robust variance-covariance estimation, to take account for repeated measurements within each woman, were used to compare sCr levels during pregnancy in women with and without preeclampsia. The sCr levels were log-transformed before entering in the model because they were not normally distributed.

Statistical analysis was carried out using SPSS v21 (SPSS Inc., Chicago, IL).

## Results

### Pregnancies

**Fig 1:**
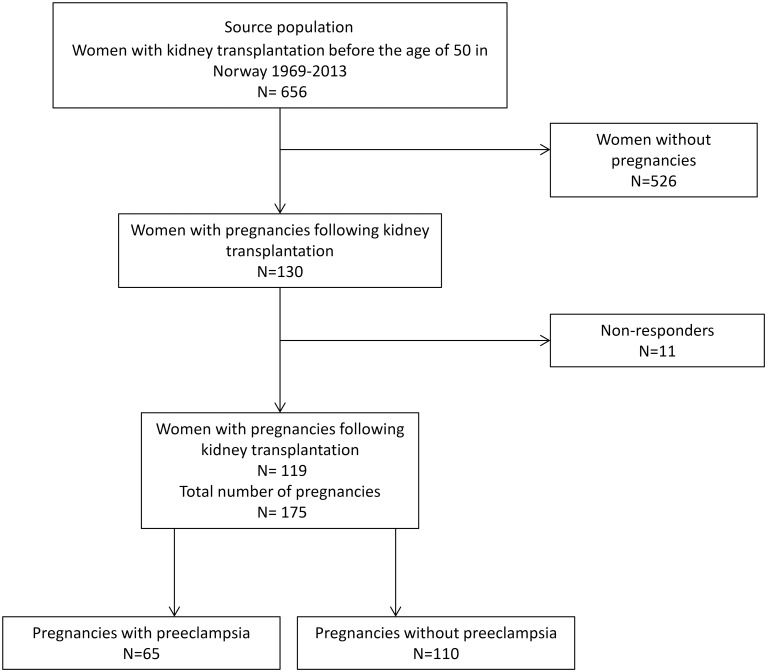
Pregnancies included in the analysis. Of 656 women in Norway with kidney transplantation before the age of 50, 130 had pregnancies following kidney transplantation. Of these, 119 consented to participate in this study.

In Norway between 1969 and 2013, 130 kidney transplanted women had pregnancies, of whom 119 consented to participate in this study (Fig 1). These women had 175 pregnancies, 65 (37.1%) of which were preeclamptic. In 29 of the 65 pregnancies (45%), women were diagnosed with preeclampsia before gestational week 34, characterized as early-onset.

Age, ethnicity, frequency of diabetes mellitus, smoking habits, and frequency of multiple pregnancies were similar between women with and without preeclampsia following kidney transplantation (Table 1). However, women with preeclampsia had higher rates of chronic hypertension than women without preeclampsia (54% vs. 17%; *P*<0.001). Previous preeclampsia in a pregnancy prior to transplantation was also more frequent in women with preeclampsia than in women without preeclampsia following transplantation (23% vs. 6%; *P* = 0.002), mean birth weight was lower (2470 ± 864 vs. 2998 ± 546 g; *P*<0.001) and mean gestational age was lower (35 ± 5 vs. 38 ± 2 weeks; *P*<0.001) in pregnancies with preeclampsia.

**Table 1:** Characteristics of pregnancies following kidney transplantation in women with and without preeclampsia.

Characteristics	Pregnancies with preeclampsia N = 65	Pregnancies without preeclampsia N = 110	*P*
Patient			
Age in years, mean (SD)	31.5 (5.0)	31.0 (5.0)	0.31
Scandinavian ethnicity, n (%)	54.0 (83)	104 (94.5)	0.05
Diabetes mellitus diagnosed before pregnancy, n (%)	5 (7.7)	4 (3.6)	0.20
Gestational diabetes, n (%)	1 (1.5)	2 (1.8)	0.69
Pre-pregnancy proteinuria, n (%)	2 (3)	1 (0.9)	0.18
Preeclampsia in a pregnancy prior to kidney transplantation, n (%)	15 (23)	7 (6)	0.002
Current smoker, n (%)	0 (0)	4 (3.6)	0.15
Immunosuppression			
Prednisolone, n (%)	63 (97)	108 (98)	0.59
Azathioprine, n (%)	45 (69)	87 (79)	0.15
Cylosporine, n (%)	42 (65)	51 (50)	0.02
Pregnancy			
Twin pregnancy, n (%)	1 (1.5)	1 (0.9)	0.61
Gestational age at birth in weeks, mean (SD)	35.0 (5.0)	38.0 (2.0)	<0.001
Birthweight in grams, mean (SD)	2470 (864)	2998 (546)	<0.001

Abbreviations: SD, standard deviation

The most common indications for kidney transplantation were glomerulonephritis (n = 59; 49.6%), pyelonephritis (n = 14; 11.8%), and diabetes (n = 9; 7.6%). The stated kidney disease did not significantly differ between preeclamptic and non-preeclamptic pregnancies. Combinations of immunosuppressive therapies and medications at the start of pregnancy differed, mostly according to era. Prednisolone was used in almost all pregnancies (n = 171; 98%), and cyclosporin was used in over two-thirds of all pregnancies (n = 125; 71%) with the majority using a combination of prednisolone, azathioprine and cyclosporine. Tacrolimus was used in 22% (n = 39) of pregnancies. Aspirin was prescribed for only three pregnancies.

### Associations between preeclampsia, allograft function and neonatal outcome

Women with preeclampsia had significantly higher sCr levels than women without preeclampsia when assessed during the first trimester of pregnancy (*P*<0.001), at delivery (*P*<0.001), and during the postpartum period (*P* = 0.004), but no difference was seen in sCr levels before pregnancy *(P* = 0.5). In women with preeclampsia, there was a four times increased risk of an increase > 20% and an eleven times increased risk of an increase > 50% in s-Cr between the first trimester and postpartum (OR 4.7 95%CI 2.2–9.8 and OR 11.6 95%CI 3.17–42.5 respectively). Episodes of acute rejection, however, were not recorded during any of the pregnancies. Graft loss within two years after delivery occurred in six (3.4%) women, four of which had preeclampsia, although the risk of graft loss was not significantly higher in women with preeclampsia than in women without it (Table 2).

**Table 2:** Association between preeclampsia and kidney function.

Characteristics		Pregnancies with preeclampsia N = 65	Pregnancies without preeclampsia N = 110	Crude OR (95% CI)	*P*
sCr (μmol/L), median (IQR)					
Before pregnancy		103 (84.5–131)	94.5 (80–108)	-	0.5
First trimester		97 (80–110)	84 (73–97)	-	0.006
At delivery		124 (99–153)	96.5 (83–116)	-	<0.001
Postpartum		108 (95–128)	90.5 (80–111)	-	0.004
Increase in sCr pre-to postpartum					
	>20%	31/52 (60%)	24/89 (27%)	4.7 (2.2–9.8)	<0.001
	>50%	15/52 (29%)	3/89 (3%)	11.6 (3.2–42.5)	<0.001
Graft loss within 2 years postpartum					
Yes		4	2	3.5 (0.6–19.9)	0.15
No		61	108		

Abbreviations: IQR, interquartile range; OR, odds ratio; sCr, serum creatinine

The risk (adjusted OR) of delivering preterm (<37 gestational weeks) was 9.2 times higher in pregnancies with preeclampsia than in pregnancies without preeclampsia (*P*<0.001). Also, the risk of delivering very preterm (<34 gestational weeks) was 10.5 times higher in pregnancies with preeclampsia than in those without it (*P*<0.001).

Eight fetal deaths (4.6%) were reported within 24 h of delivery, including two intrauterine fetal deaths and six early perinatal deaths (Table 3). The risk of fetal death in preeclamptic pregnancies was approximately three times higher than in non-preeclamptic pregnancies, although the difference was not statistically significant. However, when limited to women with hypertension despite treatment in the study population (systolic/diastolic blood pressure ≥140/90 mmHg, N = 79), this was associated with a significantly higher risk of fetal death (aOR = 9.52 [95%CI 1.10–84.70]; *P* = 0.04).

**Table 3:** Association between preeclampsia and neonatal outcome.

Characteristics		Pregnancies with preeclampsia (N = 65)	Pregnancies without preeclampsia (N = 110)	Crude OR (95% CI)	*P*	Adjusted OR^a^	*P*
Preterm delivery (<37 weeks), n (%)							
	Yes	51 (78)	32 (29)	8.88 (4.32–18.25)	<0.001	9.19 (4.44–19.02)	<0.001
	No	14 (22)	78 (71)				
Very preterm delivery (<34 weeks), n(%)							
	Yes	19 (29)	4 (4)	10.95 (3.53–33.97)	<0.001	10.55 (3.35–32.59)	<0.001
	No	46 (71)	106 (96)				
Intrauterine or perinatal fetal death within 24 h, n (%)							
	Yes	5 (8)	3 (3)	2.97 (0.69–12.87)	0.145	3.12 (0.65–14.85)	0.153
	No	60 (92)	107 (97)				
Caesarean delivery, n (%)							
	Yes	51 (78)	61 (55)	2.93 (1.45–5.90)	0.003	2.66 (1.30–5.42)	0.007
	No	14 (22)	49 (45)				
Small for gestational age (below 10^th^ percentile), n (%)							
	Yes	21 (32)	21 (19)	2.02 (1.00–4.09)	0.027		
	No	44 (68)	89 (81)				

^a^ Adjusted for age and smoking

The rate of delivery by caesarean section was high in all pregnancies following kidney transplantation (78% for women with preeclampsia vs. 56% for women without preeclampsia), although it was significantly higher for women with preeclampsia than in those without it (aOR = 2.66 [95% CI, 1.30–5.42]; *P =* 0.007). The infants born following a pregnancy with preeclampsia also more often had a birth weight below the 10^th^ percentile, defined as small for gestational age (Table 3).

### Univariate and multivariate analysis of epidemiological and clinical variables

Univariate analysis revealed chronic hypertension (*P*<0.001), preeclampsia in a pregnancy prior to transplantation (*P* = 0.002), elevated sCr (≥125 μmol/L) at the start of pregnancy (*P* = 0.025), and use of cyclosporine (*P* = 0.022) as significantly more frequent in pregnancies with preeclampsia than in those without it (Table 4). In the multivariate model, chronic hypertension (OR = 5.02 [95% CI, 2.47–10.18]; *P*<0.001), elevated sCr at the start of pregnancy (OR = 5.79 [95% CI 1.91–17.59]; *P* = 0.002), and preeclampsia in a pregnancy prior to kidney transplantation (OR = 3.26 [95% CI, 1.43–7.43]; *P* = 0.005) were identified as prognostic factors for preeclampsia.

**Table 4:** Univariate analysis of the association between preeclampsia and clinical parameters before and during pregnancy following kidney transplantation.

Characteristic		Pregnancies with preeclampsia N = 65	Pregnancies without preeclampsia N = 110	Crude OR (95% CI)	*P*
Chronic hypertension, n (%)					
	Yes	35 (54)	19 (17)	5.58 (2.79–11.19)	<0.001
	No	30 (46)	91 (83)		
Preeclampsia in a pregnancy prior to transplantation, n (%)					
	Yes	15 (23)	7 (6)	4.41 (1.69–11.51)	0.002
	No	50 (77)	103 (94)		
Elevated sCr at the start of pregnancy (≥125 μmol/L), n (%)					
	Yes	12 (18)	4 (4)	5.33 (1.24–24.79)	0.025
	No	53 (82)	106 (96)		
Diabetes mellitus, n (%)					
	Yes	5 (8)	4 (4)	3.07 (0.59–16.14)	0.18
	No	60 (92)	106 (96)		
>1 transplantation, n (%)					
	Yes	6 (9)	17 (15)	0.40 (0.09–1.90)	0.25
	No	58 (91)	93 (85)		
Twin pregnancy, n (%)					
	Yes	1 (1.5)	1 (1)	1.70 (0.11–27.70)	0.71
	No	64 (98.5)	109 (99)		
Time from transplantation to pregnancy <24 months, n (%)					
	Yes	4 (6)	7 (6)	0.97 (0.27–3.43)	0.96
	No	61 (94)	103 (94)		

Abbreviations: CI, confidence interval; sCR, serum creatinine

#### Stratification of risk

**Fig 2:**
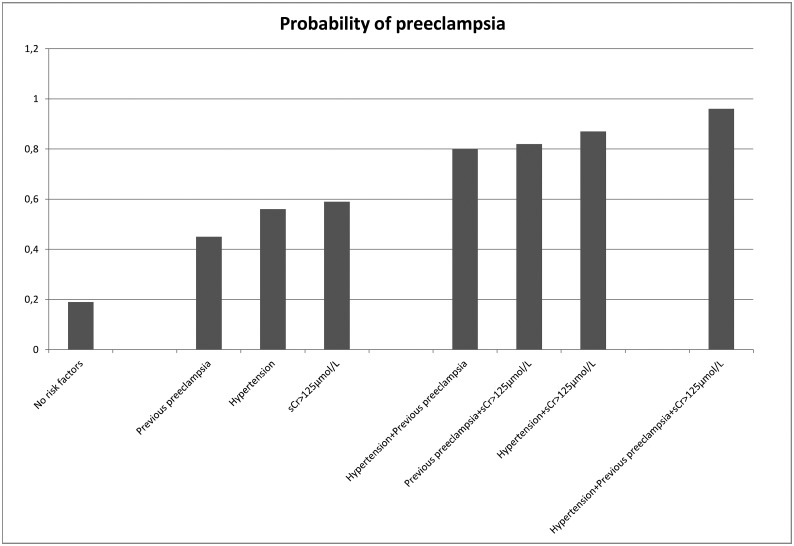
Probability of preeclampsia based on the multivariate model. Estimated probability in percentage according to the presence of different combinations of the prognostic factors.

Women without chronic hypertension, elevated sCr at start of pregnancy, or previous preeclampsia had a 19% probability of having preeclampsia (Fig 2).

The rate of preeclampsia increased to 45% to 59% when one of these factors was present. A combination of two factors increased the risk to 80% to 87%, and having all three factors increased the risk to 96% (Fig 2). The concordance index for the model was 0.70 (95% CI, 0.62–0.79).

## Discussion

In this study, we investigated the effect of preeclampsia on pregnancy outcome and allograft function in kidney transplanted women. Preeclamptic pregnancies had increased risks for preterm delivery, a small-for-gestational-age infant, and deterioration of allograft function. Based on a multivariate model, the study also showed that chronic hypertension, elevated sCr at the start of pregnancy and previous preeclampsia were significant risk factors for preeclampsia in these women and could be used to build a simple prognostic score.

Chronic hypertension is common in kidney transplanted women [12], but most studies on pregnancies following kidney transplantation have not investigated its role as a predictor of future adverse events. Our finding that chronic hypertension was a significant prognostic factor for preeclampsia in kidney transplanted women is in line with several previous reports in the general population [13]. This was not found in a recent cohort study of kidney transplanted women in the UK, although preeclampsia was not included in the definition of poor pregnancy outcomes [11].

Kidney transplanted women who had a history of previous preeclampsia had a four-fold higher risk of preeclampsia than those who were primiparous or had previous normal pregnancies. We therefore recommend that a previous history of preeclampsia be considered in pre-pregnancy consultations in kidney transplanted women and that women who have had preeclampsia have a targeted plan for close follow-up and surveillance during their pregnancies.

An elevated level of sCr (≥ 125 μmol/L) at the beginning of pregnancy was identified as a prognostic factor for preeclampsia. Preeclampsia also significantly increased the risk of elevated postpartum sCr levels, indicating possible permanent damage to the allograft. Previous reports have not shown an increased risk of graft loss in women with pregnancies following kidney transplantation [14], but the reports have either had short follow-up or have included few women, and they have not compared preeclamptic and non-preeclamptic pregnancies. Further studies with long-term follow up are needed to establish whether pregnancy is associated with future risk of graft loss in kidney transplanted women and how this may be affected by preeclampsia.

The absolute rates of perinatal mortality have never been lower in developed countries [15]. In our study, the rate of perinatal mortality in pregnancies following kidney transplantation (4.6%) was considerably higher than in the general Norwegian population (0.3%). Although our sample size was relatively small, our results suggest an association between uncontrolled hypertension and later fetal death. Jungers et al. also reported an approximately 10-fold higher relative risk of fetal death in women with mean arterial pressure >105 mmHg at conception than in women with spontaneous or therapeutically achieved normotension [16]. The association between uncontrolled hypertension and later fetal death highlights the need for aggressive management of hypertension during pregnancies in kidney transplanted women.

In previous studies, preeclampsia in kidney transplanted women has been examined as an outcome of pregnancy [2, 3, 11], whereas in the current study, we examined preeclampsia as a risk factor for pregnancy complications. An increased risk of adverse neonatal and maternal outcome in pregnancies with preeclampsia has been found in several studies in the general population and has been explained by abnormal development of the placenta and abnormal utero-placental circulation [17]. There is considerable diagnostic uncertainty in the distinction between worsening of the pre-existing disease and super-imposed preeclampsia in women with chronic kidney disease. Our study confirms a particularly high rate of early-onset preeclampsia and small-for-gestational age infants in preeclamptic pregnancies following kidney transplantation. We assume that these findings reflect possible placental insufficiency. If this finding represents true intrauterine growth restriction, it will add validity to the diagnosis of preeclampsia.

A test or score that can accurately predict preeclampsia in kidney transplanted women is yet to be available. In this study we used clinical characteristics to identify prognostic factors that can be used to determine the risk of preeclampsia. The three prognostic factors—chronic hypertension, elevated sCr at the start of pregnancy, and previous preeclampsia—can be easily determined prior to and at the beginning of pregnancy. Being able to stratify the risk for preeclampsia based on simple prognostic factors may increase early diagnosis and should help tailor clinical management and therefore improve maternal and neonatal outcome. Although all pregnant kidney transplanted women need close follow-up, this information can help physicians to adjust monitoring, prenatal visits, ultrasound evaluation, and patient education. Collecting blood pressure, sCr concentration, and accurate measurement of gestational age at the very beginning of pregnancy can help estimate the risk of preeclampsia and can help differentiate preeclampsia symptoms from symptoms of the underlying kidney disorder and assist in the differential diagnosis of fetal growth restriction.

Having a prognostic score for preeclampsia also raises the possibility of applying preventive measures, although, currently few are known. Low-dose aspirin appears to modestly reduce the risk of preeclampsia in women at high risk for perinatal death and adverse perinatal outcome [18]. Aspirin may also provide small but significant neonatal reductions in the risk of preterm delivery and intrauterine growth restriction. The Collaborative Low-dose Aspirin Study in Pregnancy, which included some women with renal disease, identified a lower rate of preterm deliveries in women treated with low-dose aspirin, indicating a shift in preeclampsia to later gestational ages [19]. Several major groups therefore recommend offering low-dose aspirin to women at high risk for preeclampsia, including women with renal disease [20, 21]. Despite these guidelines, only three of the women in our study population used aspirin. Even though aspirin has not been specifically studied in kidney transplanted women, it has been proven to be safe during pregnancy and should be considered for inclusion in the current guidelines for pregnancies following kidney transplantation and its use as a preventive treatment should be investigated in future prospective studies in this population.

Although the population included in this study was relatively homogeneous and small, it represents the total population of kidney transplanted women in Norway, with the exception of the few women who did not consent to participate. In addition, the consistent use of information from medical records and the same diagnostic criteria for the whole population strengthens the accuracy and validity of our findings and, coupled with the inclusion of all pregnancies in Norway, reduces the risks of reporting and selection bias. The risk of baseline differences in the cohort regarding nephrologic and perinatal care, socioeconomic factors and healthcare infrastructure is also thought to be minimal or non-existing.

The risk of unmeasured confounders is always present in an observational study. However, we believe that the major risk factors related to the outcomes were considered and that the accuracy of the data regarding the potential confounding factors provided valid information. Last, the relatively small size of the study limited the statistical power of comparisons, especially where rates were low, so the results must be interpreted with care and validated in an independent study.

The incidence of preeclampsia in pregnancies following kidney transplantation is known to be high, and the risk in this population has been persistent throughout the 45-year study-period [4]. The rate of early-onset preeclampsia, diagnosed before gestational week 34, is also considerably higher than in the general population.

This study demonstrated the subsequent increased risk of adverse outcomes for mother and child in preeclamptic pregnancies compared to non-preeclamptic pregnancies. To help the identification of women at high risk of preeclampsia and tailor the counseling and clinical management of pregnancies following kidney transplantation, we also provided information about prognostic factors for preeclampsia and build a simple prognostic score based on characteristics easily available at start of pregnancy. Research on the predictive value of biomarkers and their role in clinical decision making regarding preeclampsia in kidney transplanted women is promising [22], but still need further exploration. Hopefully will this present study and the increased knowledge about preeclampsia in kidney transplanted women, also be of value for prospective future studies on therapeutic intervention and prevention of preeclampsia in kidney transplanted women.
